# Gene expression derived from alternative promoters improves prognostic stratification in multiple myeloma

**DOI:** 10.1038/s41375-021-01263-9

**Published:** 2021-05-10

**Authors:** Luis V. Valcárcel, Ane Amundarain, Marta Kulis, Stella Charalampopoulou, Ari Melnick, Jesús San Miguel, José I. Martín-Subero, Francisco J. Planes, Xabier Agirre, Felipe Prosper

**Affiliations:** 1 Area de Oncología, Centro de Investigación Médica Aplicada (CIMA), Universidad de Navarra, IDISNA, Pamplona, Spain; 2 Tecnun School of Engineering, Universidad de Navarra, San Sebastian, Spain; 3 Centro de Investigación Biomédica en Red de Cáncer, CIBERONC, Pamplona, Spain; 4 Fundació Clínic per a la Recerca Biomèdica, Barcelona, Spain; 5 Institut d’Investigacions Biomèdiques August Pi I Sunyer (IDIBAPS), Barcelona, Spain; 6 Division of Hematology/Oncology, Department of Medicine, Weill Cornell Medical College, New York, NY USA; 7 Servicio de Hematología, Clínica Universidad de Navarra, Pamplona, Spain; 8 Institució Catalana de Recerca i Estudis Avançats (ICREA), Barcelona, Spain; 9 Departamento de Fundamentos Clínicos, Universitat de Barcelona, Barcelona, Spain

**Keywords:** Myeloma, Risk factors

## Abstract

Clinical and genetic risk factors are currently used in multiple myeloma (MM) to stratify patients and to design specific therapies. However, these systems do not capture the heterogeneity of the disease supporting the development of new prognostic factors. In this study, we identified active promoters and alternative active promoters in 6 different B cell subpopulations, including bone-marrow plasma cells, and 32 MM patient samples, using RNA-seq data. We find that expression initiated at both regular and alternative promoters was specific of each B cell subpopulation or MM plasma cells, showing a remarkable level of consistency with chromatin-based promoter definition. Interestingly, using 595 MM patient samples from the CoMMpass dataset, we observed that the expression derived from some alternative promoters was associated with lower progression-free and overall survival in MM patients independently of genetic alterations. Altogether, our results define cancer-specific alternative active promoters as new transcriptomic features that can provide a new avenue for prognostic stratification possibilities in patients with MM.

## Introduction

Multiple myeloma (MM) is a hematological malignancy characterized by an abnormal accumulation of clonal plasma cells (PC) in the bone marrow. In recent years, the survival of MM patients has increased significantly [1] but, regrettably, MM is still considered an incurable disease [2]. Given the underlying heterogeneity of MM, and in spite of the clinical value of genetic alterations [3], the discovery of novel biomarkers to further improve its prognostic stratification remains challenging. Several studies have attempted to address this issue using different high-throughput-technologies [3]. While the study of the transcriptome using RNA-seq is very common in cancer research, it is not currently applied as a risk stratification tool in patients with MM [4].

Recently, Demircioğlu et al. [5] presented a novel approach to study transcriptome regulation in cancer cells, defining gene active promoters (AP) from RNA-seq data and showing that expression derived from alternative active promoters (AAP) can be used as a biomarker to improve the stratification of cancer patients. Inspired by this approach, we have exploited RNA-seq data from B-cells and MM patients to investigate the possible role of AAP as a new prognostic biomarker for improving the survival stratification of MM patients.

## Methods

### Promoter activity definition

We used the R package *proActive* to identify promoters within the annotated genes in Gencode v27. Moreover, we used STAR v 2.6.1a and our strand-specific RNA-seq (ssRNA-seq) data from PC of MM patient samples (*n* = 32) and normal B-cells (*n* = 35), to identify AP (defined as the transcription amount initiated from each of the promoters related to one specific gene), and AAP (APs showing alternative usage in different conditions) in each B-cell subpopulations and PC of MM patient samples. All identified promoters were also correlated with different epigenetic marks and the genome segmentation into chromatin states in MM and B-cell populations, as described in a previous study from our group [6]. Further details are shown in Supplemental Methods.

### Survival analyses

Survival analyses were performed with the IA14 release data of the multiple myeloma research foundation (MMRF) CoMMpass Study dataset (595 samples). Please be referred to Supplemental Methods for further information.

## Results and discussion

**Fig. 1:**
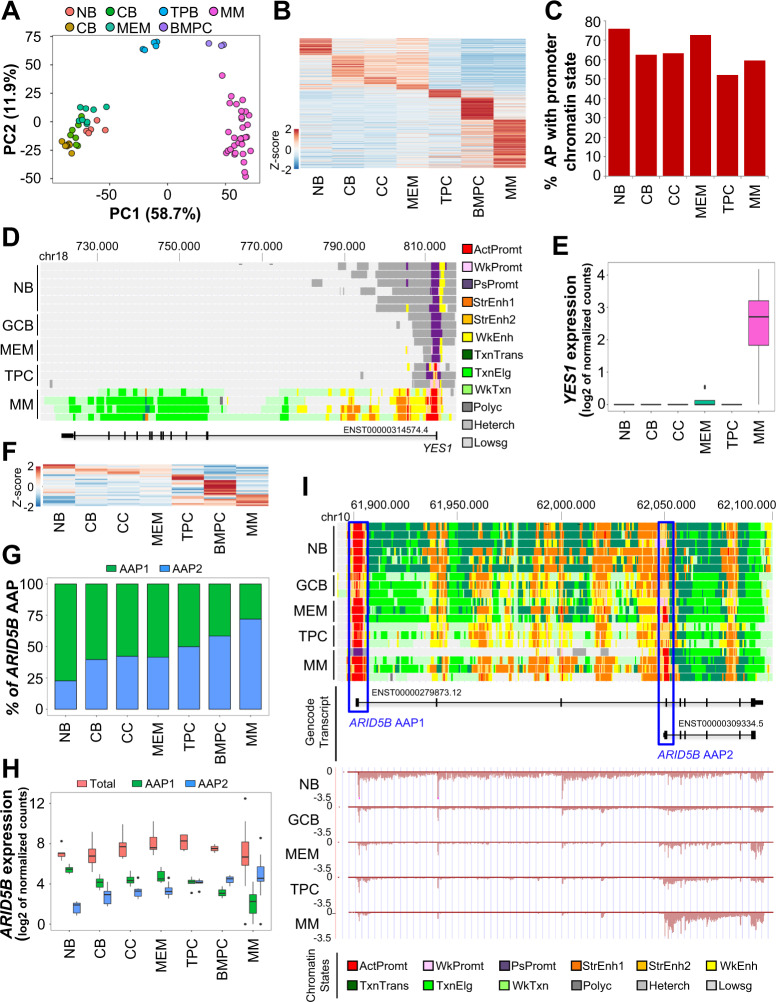
Active promoters and alternative active promoters in B cell subpopulations and MM patient samples. **A** PCA of the absolute promoter activity of the top 1500 promoters with the highest variance. **B** Heatmap showing the mean absolute promoter activity for cell-specific active promoters. Data were scaled across cell types for visualization. **C** Percentage of AP with promoter chromatin state in each cell type. **D** Genome browser snapshot showing chromatin states and de novo active promoter of *YES1* locus in normal B cell subpopulations and MM patients. **E** *YES1* expression levels in B cells subpopulations and MM patient samples. Gene expression is showed in log2 of normalized counts. **F** Heatmap showing the mean absolute promoter activity for cell-specific alternative promoters (AAP) of genes, which do not change in overall gene expression across cell types. Data were scaled across cell types for visualization. **G** Percentage of activity of *ARID5B* alternative promoters (AAP1 and AAP2), explaining the contribution of each promoter to *ARID5B* total gene expression in each B cell subset. **H** Total *ARID5B* expression and its expression derived from each of the two alternative promoters (AAP1 and AAP2) in each B cell subset. Absolute promoter activity and gene expression are in log2 of normalized counts. *ARID5B* gene expression does not change throughout all B cell differentiation and MM, however, the principal promoter shifts from AAP1 to AAP2 during the differentiation process and the shift to MM. **I** Genome browser snapshot showing chromatin states (up panel) and expressed alternative transcripts (below panel) from *ARID5B* locus in normal B cell subpopulations and MM patients. Transcript ID is indicated below the transcript scheme. NB: naïve; GCB: germinal center; CB: centroblast; CC: centrocyte; MEM: memory B cell; TPC: tonsil plasma cell; BMPC: bone marrow plasma cell; MM: plasma cells of MM patient samples; AAP: alternative active promoter. Chromatin States: ActPromt: active promoter; WkPromt: weak promoter; PsPromt: poised promoter; StrEnh1: strong enhancer 1; StrEnh2: strong enhancer 2; WkEnh: weak enhancer; TxnTrans: transcription transition; TxnElg: transcription elongation; WkTxn: weak transcription; Polyc: polycomb; Heterch: heterochromatin; Lowsg: low signal.

Based on the strategy described by Demircioğlu et al. [5], we initially defined the AP in different B-cell subpopulations including normal PCs from healthy donors and PCs from MM patients taking the Gencode v27 annotation as reference [7]. We identified 115,496 possible promoters and defined their activity using our ssRNA-seq data by quantifying the expression that is initiated at each promoter for each B cell subpopulation and PC, therefore identifying AP in each specific subpopulations (Supplemental data 1). Using this promoter activity, principal component analysis (PCA) revealed robust segregation of PC from the rest of B-cells including a clear distinction between normal and MM PC (Fig. 1A). Furthermore, differential analyses revealed the presence of AP specific for each B-cell subpopulation and MM PC (Fig. 1B). These results are consistent with our previous results using global gene expression [6, 7].

The accuracy of promoter activity was validated using our previously described chromatin data generated from the same populations of B cells and PC including H3K4me3 and H3K27Ac ChIP-seq data, histone marks associated with AP [6, 8]. RNA-based estimates from patient samples accurately reflect the promoter activity, as 50–75% of the APs detected in each cell subpopulation show chromatin states indicative for AP (Fig. 1C). Focusing on MM, we observed that some of these promoters were de novo AP (Fig. 1D, E), i.e., AP associated with genes expressed only in MM and not in normal B-cells, such as *PRDM5*, *IGF1*, or *BMP6*, among others, as we recently described [6]. These results indicate that expression and chromatin-based estimates are consistent and therefore, RNA-seq can provide, in addition to gene expression, an important estimation of the chromatin states and promoters location from which gene transcription is taking place in each cell type.

The presence of AAP in cancer has recently been associated with the transcription of different gene isoforms [5]. Interestingly, we observed that the expression of AAP was also specific to MM and B-cell subsets and that it also correlated with epigenomic data (Fig. 1C, F, Supplemental data 2). Such is the case of *ARID5B*, a gene that shows a shift in promoter usage throughout B cell differentiation and MM (Fig. 1G–I).

**Fig. 2:**
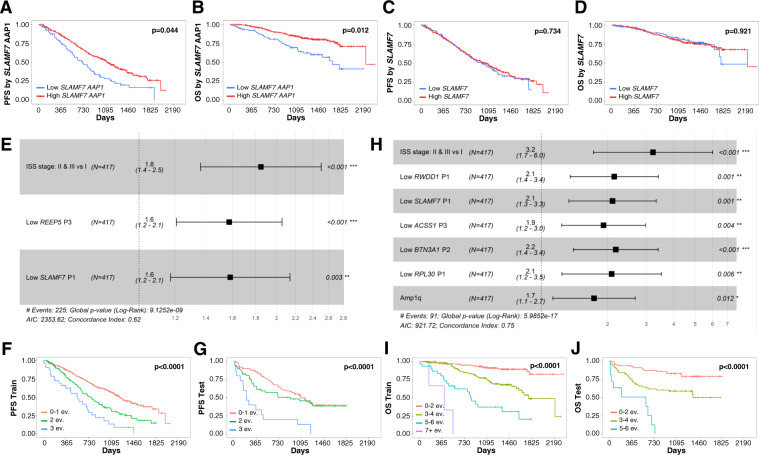
AAP definition improves the prognosis stratification of MM patients. **A**, **B** PFS and OS stratification by *SLAMF7* promoter 1 activity. **C**, **D** PFS and OS stratification by *SLAMF7* gene expression activity. **E** Final model selected with BIC for PFS including the activity of the AAP1 of *SLAMF7*, AAP3 of *REEP5*, and the ISS stage. **F**, **G** Kaplan–Meier curves with dichotomized events of PFS in training and test set. The full figure is shown in Supplemental Fig. 1. **H** Final model selected with BIC for OS including the activity of the AAP2 of *BTN3A1*, AAP1 of *RPL30*, AAP3 of *ACSS1*, AAP1 of *RWDD1*, AAP1 of *SLAMF7*, ISS stage, and amplification 1q21. **I**, **J** Kaplan–Meier curves with dichotomized events and grouped for better visualization of OS in training and test set. The full figure is shown in Supplemental Fig. 2. Number of events: refers to those factors included in the final PFS or OS model, respectively. ev: events.

The role of the transcriptional profile in the prognosis of MM has been clearly demonstrated and specific transcriptional signatures have been associated with specific subgroups of MM patients [9]. Based on our previous analysis, we hypothesized that expression of AAP could represent a new prognostic factor in MM, as different isoforms of the same genes may contribute to distinct clinical impact. To address this question we took advantage of the RNA-seq data of the CoMMpass study that includes 595 MM patient samples acquired at diagnosis. Following the AAP selection criteria defined by Demircioğlu et al. [5], we identified 1539 AAP within MM patient samples. We divided the cohort of MM patients into training and test datasets. Using the training data, we performed a univariate coxph analysis for each AAP in both PFS and OS (Supplemental methods). The expression derived from 6 AAP within MM patient samples was significantly associated with PFS, and 18 AAP with OS. To avoid bias derived from differentially expressed genes, we kept AAP whose associated gene expression did not show statistical significance for PFS and/or OS, which yielded a final list of 3 AAP significantly correlated with PFS and 10 AAP with OS of MM patients (Supplemental data 3). As an example, while *SLAMF7* expression was not associated with PFS or OS, a higher activity of AAP1 of *SLAMF7* was associated with improved PFS and OS in MM patients (Fig. 2A–D). These results indicate that assessment of AAP may contribute to identifying new prognostic factors.

Next, using multivariate Cox regression model and stepwise model selection with Bayesian information criterion (BIC) [10], we evaluated the hazard prediction power, for both PFS and OS, of the combination between AAP and risk genetic markers in MM patients [11–15]: ISS, t(4;14), t(14;16), t(14;20), del17p, deletion of *CDKN2C*, del1p, amp1q and mutations of *TP53*. Interestingly, we detected that AAP significantly over-perform the information provided by genetic alterations in terms of PFS and OS. In the multivariate analysis, we identified ISS stage and AAP of *REEP5* and *SLAMF7* genes (Supplemental Fig. 1, Supplemental data 4) with independent prognostic value for PFS, and provided a predictive model whose cumulative activity discriminated 3 different risk groups (Fig. 2E, F). This was also validated in the test cohort (Fig. 2G). Regarding OS, we identified the AAP of five genes, *RWDD1*, *SLAMF7*, *ACSS1*, *BTNG3A1*, and *RPL30* (Supplemental Fig. 1, Supplemental data 4) that together with the ISS stage and amplification of 1q discriminated patients with different prognosis (Fig. 2H; Supplemental Fig. 2A). Again, these factors generated a risk assessment model with four distinct risk groups for OS (Fig. 2I) in the training cohort that was validated in the test cohort (Fig. 2J; Supplemental Fig. 2B). Finally, we also performed an ANOVA test to compare the models derived from genetic risk factors only or combining genetic risk factors with AAP, finding a significant improvement for the combination of both risk factors for PFS (*p*-value = 2e–5) and OS (*p*-value = 4e–10).

In summary, in this study, we demonstrate that RNA-seq data can be exploited in a non-conventional way to identify AP and AAP in MM and that the expression derived from AAP shows a greater contribution as a survival risk biomarker than high-risk genetic classifiers used currently in the clinical outcome of MM patients.

## Supplementary information

Supplemental Figures

Supplemental material

Supplemental data 1

Supplemental data 2

Supplemental data 3

Supplemental data 4
