# Supplementary material for: Gene expression derived from alternative promoters improves prognostic stratification in multiple myeloma
Source: Leukemia. 2021 May 10;35(10):3012–6. doi: 10.1038/s41375-021-01263-9 (PMC8478642; doi:10.1038/s41375-021-01263-9)
Supplement: Supplementary file 1 — Supplemental Figures [file 41375_2021_1263_MOESM1_ESM.pptx]

## Slide 1
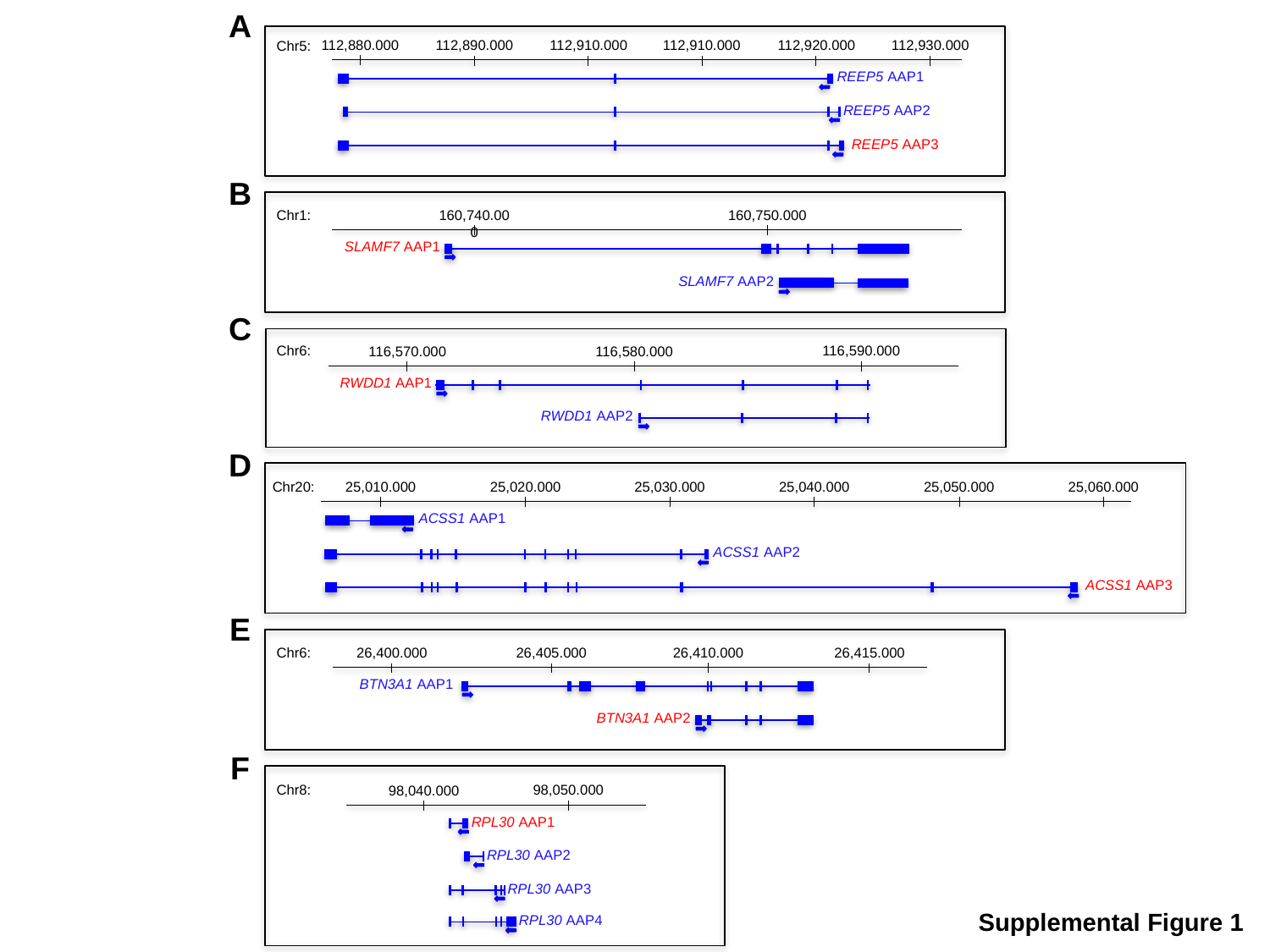

A
112,880.000
112,890.000
112,910.000
112,910.000
112,920.000
112,930.000
Chr5:
REEP5 AAP1
REEP5 AAP2
REEP5 AAP3
B
160,740.000
160,750.000
Chr1:
SLAMF7 AAP1
SLAMF7 AAP2
C
Chr6:
116,590.000
116,570.000
116,580.000
RWDD1 AAP1
RWDD1 AAP2
D
25,030.000
25,060.000
Chr20:
25,010.000
25,020.000
25,040.000
25,050.000
ACSS1 AAP1
ACSS1 AAP2
ACSS1 AAP3
E
26,410.000
Chr6:
26,400.000
26,405.000
26,415.000
BTN3A1 AAP1
BTN3A1 AAP2
F
98,050.000
Chr8:
98,040.000
RPL30 AAP1
RPL30 AAP2
RPL30 AAP3
RPL30 AAP4
Supplemental Figure 1

## Slide 2
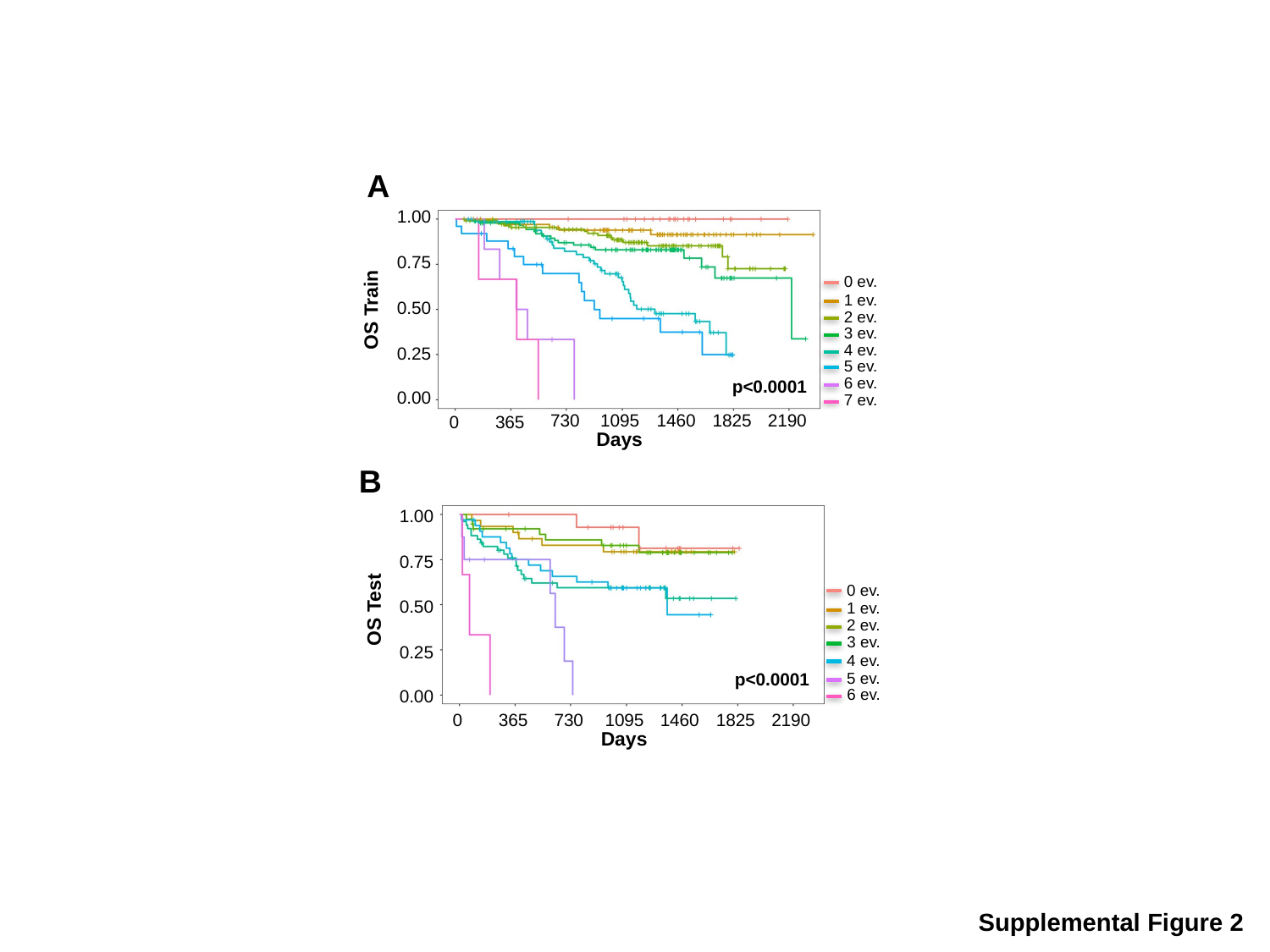

A
1.00
OS Train
0.75
0 ev.
1 ev.
2 ev.
3 ev.
4 ev.
5 ev.
6 ev.
7 ev.
0.50
0.25
p<0.0001
0.00
730
1095
1460
1825
2190
0
365
Days
B
1.00
OS Test
0.75
0 ev.
1 ev.
2 ev.
3 ev.
4 ev.
5 ev.
6 ev.
0.50
0.25
p<0.0001
0.00
0
365
730
1095
1460
1825
2190
Days
Supplemental Figure 2
