# Supplementary material for: Gene expression derived from alternative promoters improves prognostic stratification in multiple myeloma
Source: Leukemia. 2021 May 10;35(10):3012–6. doi: 10.1038/s41375-021-01263-9 (PMC8478642; doi:10.1038/s41375-021-01263-9)
Supplement: Supplementary file 2 — Supplemental material [file 41375_2021_1263_MOESM2_ESM.docx]

**SUPPLEMENTAL METHODS**

**Samples and Strand-Specific RNA (ssRNA-seq) sequencing**

Bone marrow aspiration samples were obtained from 33 newly diagnosed untreated MM patients. Plasma cells were purified by CD138+ positive magnetic separation using AutoMACS system (AutoMACS Pro Separator, Miltenyi Biotech), obtaining >90% purity in all cases confirmed by flow cytometry. The data from 35 samples of different normal B-cells (Naive, memory, centroblast, centrocytes, tonsil plasma cells and bone marrow plasma cells) was generated by our group using strand specific RNA-sequencing (ssRNA-seq) as previously described^1^. ssRNA-seq data of B cell subpopulations and MM patients are available at GEO under accession numbers GSE114816 and GSE151063. All patients and healthy donors gave informed consent for their participation in this study, which was approved by the clinical research ethics committee of Clínica Universidad de Navarra.

**Study of promoters and alternative promoters in each cell type**

Active Promoters (AP) and Active Alternative Promoters (AAP) values were calculated following the approach described by Demircioglu et al. 2019. We used R proActive package, where the units correspond to log2 normalized counts from DESeq2^2^, an standard approach for measuring gene expression with RNA-seq.

For each promoter *p* in sample *i*, we have the absolute promoter expression $A_{pi}$ that is measured in log2 normalized DESeq2 counts; the relative promoter expression $R_{pi}$, which represents the contribution of each specific promoter to the total gene expression ($G_{pi}$) in a particular sample.

We investigated active and alternative promoters using the following linear model:

$$A_{pi}= \sum_{\forall j} \beta_{pj}^{A}*X_{ij}$$

$$R_{pi}= \sum_{\forall j} \beta_{pj}^{R}*X_{ij}$$

$$G_{pi}= \sum_{\forall j} \beta_{pj}^{G}*X_{ij}$$

Where $X_{ij}=1$ if a sample *i* belongs to a particular subpopulation *j,* 0 otherwise;$\beta_{pj}^{A}$, $\beta_{pj}^{R}$ and $\beta_{pj}^{G}$ store the mean absolute promoter expression, mean relative promoter expression and mean gene expression for subpopulation *j (j=1,…,n)*.

P-values and fold-changes were calculated using the following statistical contrast:

$$\left\{ \begin{matrix} {H_{0}: \beta}_{pk}-\frac{\sum_{\forall j/j\neq k} \beta_{pj}}{n-1}=0 \\ H_{1}: \beta_{pk}-\frac{\sum_{\forall j/j\neq k} \beta_{pj}}{n-1}\neq0 \end{matrix} \right.$$

, where *n* is the number of tissues. This analysis was conducted for different promoters and sub-populations using *Limma*^3^ package in R.

Selected cell-specific active promoters must satisfy: 1) FDR adjusted p-value lower than 0.05; 2) fold-change greater than 2 in absolute promoter expression and greater than 1.2 in relative promoter expression. In addition, an average expression across all samples greater than 0.1 and 0.05 is required for absolute and relative promoter expression, respectively, and an average threshold of 1 and 0.25 for the cell-specific samples.

For cell-specific alternative promoters, due to the limited number of samples, we diminished the thresholds for relative promoter expression, requiring a fold-change greater than 1.05 and FDR adjusted p-value < 0.1. In addition, associated genes must have a fold-change lower than 1.5. In addition, an average expression across all samples greater than 0.25 and 0.1 is required for absolute and relative promoter expression, respectively, and an average threshold of 0.75 for gene expression.

**Chromatin histone marks analysis**

To investigate the chromatin landscape of promoters identified from ssRNA-seq data in distinct B cell subpopulations and MM patient samples, we employed the epigenetic data and the genome segmentation into chromatin states of different B-cell subpopulations and MM patient samples described in Ordoñez *et al*^4^. Particularly, in order to evaluate the accuracy of promoter activity, we analyzed the chromatin states within the region of 500bp upstream and downstream from TSS of genes with cell type-specific APs defined by RNA-seq. We calculated the percentages of APs that contained Active Promoter or Promoter-related Enhancer states (Strong Enhancer1 from Ordoñez et al.) in at least one sample in distinct B cell subpopulations and MM patient samples. From this analysis, we excluded BMPC-specific APs, as we do not possess the chromatin states data of this normal cell type.

**Survival analyses**

For survival analyses, data from 595 MM patients included in the IA14 release of the Multiple Myeloma Research Foundation (MMRF) CoMMpass Study dataset were used. CoMMpass sequencing data has been accessed in **dbGaP** with the **Study Accession:** phs000748.v7.p4. PFS and OS information is stored in the MMRF web portal (<https://research.themmrf.org> and [www.themmrf.org](http://www.themmrf.org)). The central 95% of samples were selected according to read number classification and samples with a median quality of <30 per base were filtered^5^, selecting 595 MM samples with available clinical information for both progression-free (PFS) and overall survival (OS), ISS stage and risk genetic markers. After mapping the RNA-seq to Gencode (v27)^5^ using STAR v 2.6.1a^6^, we filtered promoter expression using the criteria proposed by Demircioğlu *et al*^7^: 1) selected promoters must be associated to a gene with two or more promoters with > 10 junction reads per sample, 2) promoter relative activity must be > 0.5 in a minimum of 105 patients.

We separated MM patient sample CoMMpass dataset into two subsets: training (70%) and test (30%). For each patient, we collected the genetic risk prognostic factors data: ISS, t(4;14), t(14;16), t(14;20), del17p, del*CDKN2C*, del1p, amp1q and mutations of *TP53*; as well as the activity of promoters selected above. We used the *caret* package in R in order to obtain a homogeneous distribution of genetic risk prognosis factors^6–10^ in the training and test set. Subsequently, we dichotomized the activity of these selected promoters and the expression level of related genes into high and low levels. To determine the promoter activity and gene expression cutoff values, we used the *maxstat* package in *R*, which maximizes the logRank cutoff value. In order to avoid bias in the definition of the expression of cutoff values, we calculated these cutoff values in 5000 random runs, each with one third of the samples, in both PFS and OS. For each run, we calculated the mean cutoff values for PFS and OS^11,12^; and finally, we used the median of these mean cutoff values across the 5000 runs. Note here that these activity cutoff values were calculated with training data.

Univariate analysis was performed using Cox regression survival analysis, for PFS and OS of MM patients taking into account each of the AAP detected in MM patients which surpasses initial thresholds and all the MM risk genetic biomarkers mentioned above: ISS (1 vs 2 and 3), t(4;14), t(14;16), t(14;20), deletion of *CDKN2C*, deletion of 17p, deletion of 1p, amplification of 1q and mutations in *TP53*. All p-values of the univariate analysis were corrected using FDR.

AAP and risk genetic biomarkers with a statistically significant prognostic value for PFS or OS in the univariate analysis were combined in a multivariate analysis, selecting the complexity of the model using the Bayesian information Criteria (BIC). This methodology only includes variables in the final model if they provide more information than the cost of adding more variables in the model. We chose BIC over AIC (Akaike information Criteria) because it produces a more sparse model, which improves the applicability in clinical practice and increases the understanding of the model.

REFERENCES

1. Agirre X, Meydan C, Jiang Y, Garate L, Doane AS, Li Z, et al. Long non-coding RNAs discriminate the stages and gene regulatory states of human humoral immune response. *Nat Commun*. 2019;10(1).

2. Love MI, Huber W, Anders S. Moderated estimation of fold change and dispersion for RNA-seq data with DESeq2. *Genome Biol*. 2014;15(12):550.

3. Ritchie ME, Phipson B, Wu D, Hu Y, Law CW, Shi W, et al. Limma powers differential expression analyses for RNA-sequencing and microarray studies. *Nucleic Acids Res*. 2015;43(7):e47.

4. Ordoñez R, Kulis M, Russiñol N, Chapaprieta V, Carrasco-Leon A, García-Torre B, et al. Chromatin activation as a unifying principle underlying pathogenic mechanisms in multiple myeloma. Genome Res. 2020;30(9):1217–1227.

5. Andrews S, Krueger F, Elix, et al. {FastQC}. January 2012.

6. Braggio E, Kortüm KM, Stewart AK. SnapShot: Multiple Myeloma. *Cancer Cell*. 2015;28(5):678-678.e1.

7. Walker BA, Mavrommatis K, Wardell CP, Ashby TC, Bauer M, Davies F, et al. A high-risk, Double-Hit, group of newly diagnosed myeloma identified by genomic analysis. *Leukemia*. 2019;33(1):159-170.

8. Binder M, Rajkumar S V, Ketterling RP, Greipp PT, Dispenzieri A, Lacy MQ, et al. Prognostic implications of abnormalities of chromosome 13 and the presence of multiple cytogenetic high-risk abnormalities in newly diagnosed multiple myeloma. *Blood Cancer J*. 2017;7(9).

9. Qazilbash MH, Saliba RM, Ahmed B, Parikh G, Mendoza F, Ashraf N, et al. Deletion of the Short Arm of Chromosome 1 (del 1p) is a Strong Predictor of Poor Outcome in Myeloma Patients Undergoing an Autotransplant. *Biol Blood Marrow Transplant*. 2007;13(9):1066-1072.

10. Tomas P, Miroslava V, Jiri M, et al. Jana B, Jaroslav B, Marie J, et al. Translocation t(8;14) in multiple myeloma defines patients with very poor prognosis-single centre experience. *Clin. Lymphoma, Myeloma Leuk.* 2015;15:e122.

11. Seckinger A, Meissner T, Moreaux J, Depeweg D, Hillengass J, Hose K, et al. Clinical and prognostic role of annexin A2 in multiple myeloma. *Blood*. 2012;120(5):1087-1094.

12. Delgado J, Pereira A, Villamor N, López-Guillermo A, Rozman C. Survival analysis in hematologic malignancies: Recommendations for clinicians. *Haematologica*. 2014;99(9):1410-1420.

**SUPPLEMENTAL FIGURE LEGENDS**

**Supplemental Figure 1:** Schematic representation of transcripts generated from each AAP described for the 6 genes (A) REEP5; B) SLAMF7; C) RWDD1; D) ACSS1; E) BTN3A1 and F) RPL30) significantly associated with the outcome of MM patients. Only one representative transcript has been depicted for each AAP, however, as reviewed in Supplemental data 4, multiple transcripts with different functional outcomes can be transcribed from each AAP. The AAPs in each gene associated with the survival of MM patients have been marked in red. Transcript structure information has been obtained from Ensembl GRCh38.p13.

**Supplemental Figure 2: AAP definition improves the prognostic stratification of MM patients. (A-B)** Kaplan-Meier curves with all defined events showing OS in the **(A)** Training and **(B)** Test Set. Number of events: number of factors included in the final PFS or OS model, respectively.

**SUPPLEMENTAL DATA**

**Supplemental data 1: Active promoters (AP) defined in each B cell subpopulation and MM patients.** Log-Fold Change and p-values correspond to the comparison of the promoter activity in the selected population vs the mean activity of the other populations. Transcript ID column gathers all transcripts that are expressed from each specific gene promoter. NB: Naïve; CB: centroblast; CC: centrocyte; MEM: memory B cells; TPC: tonsillar plasma cells; BMPC: bone marrow plasma cells and MM: multiple myeloma patient samples.

**Supplemental data 2: Alternative Active promoters (AAP) defined in each B cell subpopulation and MM patients.** Log-Fold Change and p-values correspond to the comparison of the promoter activity in the selected population vs the mean activity of the other populations. Transcript ID column gathers all transcripts that are expressed from each specific gene promoter. NB: Naïve; CB: centroblast; CC: centrocyte; MEM: memory B cells; TPC: tonsillar plasma cells; BMPC: bone marrow plasma cells and MM: multiple myeloma patient samples.

**Supplemental data 3: AAP significantly correlated with PFS or OS of MM patients.** Univariate *coxph* analysis was performed for both PFS and OS, using promoter absolute expression and gene expression. P-values were normalized using FDR. If the promoter was added to the multivariate analysis and if they were added to the final model using BIC (Bayesian Information Criteria), is showed. An additional table indicates the prognostic value of high-risk genetic factors. All the analyses were performed in the training set, using only 70% of the 595 patients.

**Supplemental data 4: List of RNA transcripts and protein isoforms derived from each promoter of the 6 genes whose AAPs have been included in the mutivariate survival predictive models.** REEP5 has been included in the PFS model; ACSS1, BTN3A1, RPL30 and RWDD1 have been included in the OS model; and SLAMF7 in both models. Gene, transcript and protein IDs have been obtained from Gencode 27.
